# Health related quality of life in patients with diabetic foot ulceration — translation and Polish adaptation of Diabetic Foot Ulcer Scale short form

**DOI:** 10.1186/s12955-017-0587-y

**Published:** 2017-01-21

**Authors:** Tomasz Macioch, Elżbieta Sobol, Arkadiusz Krakowiecki, Beata Mrozikiewicz-Rakowska, Monika Kasprowicz, Tomasz Hermanowski

**Affiliations:** 1 Department of Pharmacoeconomics, Medical University of Warsaw, Żwirki i Wigury 81, 02-091 Warszawa, Poland; 2 Medical University of Warsaw Central Clinical Hospital, Banacha 1a, 02-097 Warszawa, Poland; 3 PODOS Wound Healing Clinic, Narbutta 46/48, 02-541 Warszawa, Poland; 4 Department of Gastroenterology and Metabolic Diseases, Medical University of Warsaw, Banacha 1a, 02-097 Warszawa, Poland

## Abstract

**Objectives:**

Diabetic foot ulcer (DFU) is a common complication of diabetes and not only an important factor of mortality among patients with diabetes but also decreases the quality of life. The short form of Diabetic Foot Ulcer Scale (DFS-SF) provides comprehensive measurement of the impact of diabetic foot ulcers on patients’ health related quality of life (HRQoL). The purpose of this study was to translate DFS-SF into Polish and evaluate its psychometric performance in patients with diabetic foot ulcers.

**Methods:**

The DFS-SF translation process was performed in line with Principles of Good Practice for the Translation and Cultural Adaptation Process for patient reported outcome measures (PROMs) developed by ISPOR TCA group. Assessment of the reliability and validity of Polish DFS-SF was performed in native Polish patients with current DFU.

**Results:**

The DFS-SF validation study involved 212 patients diagnosed with DFU, with 4.4 years of DFU duration on average. The average ulcer size was 5.5 sq. cm, and generally only one limb was affected. Men (72%) and type 2 diabetes patients (86%) prevailed, with 17.8 years representing the mean time since diagnosis. The mean population age was 62.5 years. The internal consistency of all scales of the Polish DFS-SF was high (Cronbach’s alpha ranged from 0.82 to 0.93). Item convergent and discriminant validity was satisfactory (median corrected item-scale correlation ranged from 0.61 to 0.81). The Polish DFS-SF demonstrated good construct validity when correlated with the SF-36v2 and showed better psychometric performance than SF-36v2.

**Conclusions:**

The newly translated Polish DFS-SF may be used to assess the impact of DFU on HRQoL in Polish patients.

**Electronic supplementary material:**

The online version of this article (doi:10.1186/s12955-017-0587-y) contains supplementary material, which is available to authorized users.

## Introduction

Diabetic foot ulcer (DFU) is a common complication of diabetes — it is estimated up to 15–25% of all patients with diabetes will experience ulceration of the foot during their lifetime [1, 2]. Recent studies have showed easy accessible assessment of the progression of diabetic retinopathy by ophthalmological examination [3]. It is a reliable indicator of the perfusion defects in the lower limbs. However diabetic foot syndrome is still diagnosed late and ulceration of foot is the main cause of lower extremity of amputation in diabetes and a major determinant of disability [4]. Diabetic foot syndrome is not only an important factor of mortality among patients with diabetes but also decreases quality of life (QoL) [5, 6]. Indeed several trials showed that patents with foot ulceration have significantly decreased health related quality of life (HRQoL) compared to those without this complication. Valensi et al. found that HRQoL measured with SF-36 was significantly lower for all domains in a group of patients with foot ulcers compared to those without foot ulcers [7]. Ribu et al. found that the patients with diabetic foot ulcer reported significantly poorer HRQoL than the diabetes population in all the SF-36 subscales and in the both SF-36 summary scales [8]. In another study, Ribu et al. found that after 12 months of observation, subjects with ulcers that did not heal had HRQoL significantly lower than that of subjects with healing ulcers in five of eight subscales in the SF-36 [9]. Moreover, Winkley et al. found that the quality of life deteriorates if foot ulcer recurs or does not heal [10]. Most of cited studies used SF-36 for quality of life measures and although SF-36 has shown sensitivity when correlating HRQoL scores with diabetic foot ulcers severity some study question its sensitivity to ulcer healing [11, 12]. It is suggested that SF-36 measures of HRQoL may be confounded by non-foot complications of diabetes [8, 11]. In order to overcome those potential confounding factors, a variety of condition— and region-specific Patient-Reported Outcome Measures (PROMs) were used to assess HRQoL in patients with diabetic foot ulcer [13]. The Diabetic Foot Ulcer Scale (DFS) and short form of the DFS (DFS-SF) provides comprehensive measurement of the impact of diabetic foot ulcers on patients’ QoL [14, 15]. The Diabetic Foot Ulcer Scale consists of 58 items (each on a 5-point Likert-type scale) grouped into 11 domains used to compute 15 QoL subscales: leisure, physical health, medicine effect, daily life, dependence, emotions, healthy behaviors, medical compliance, family life, friends, ulcer care, satisfaction, personal care, positive relationship and the financial burden [14]. The shorter form of the DFS, the DFS-SF contains a total of 29 items (each on a 5-point Likert-type scale) comprising six subscales: leisure, physical health, dependence/daily life, negative emotions, worried about ulcers/feet and bothered by ulcer care [15]. This short form of the DFS was developed to reduce patient burden and proved to have good psychometric properties DFS-SF (original language English) has been translated to several languages including Chinese, Dutch, French, Mandarin and Spanish [16]. However, only the Chinese translation has undergone a full linguistic validation process [17].

To the best of our knowledge, the HRQoL in the population of patients with DFU in Poland has not been previously analyzed. Moreover, translated condition— and region-specific PROMs that assess HRQoL in patients with diabetic foot ulcer are not currently available in Poland. The aim of our study was to translate DFS-SF into Polish and evaluate its psychometric performance. Secondary objectives of this study were to investigate the influence of severity of foot ulceration on HRQoL.

## Methods

The DFS-SF translation process was performed in line with Principles of Good Practice for the Translation and Cultural Adaptation Process for Patient-Reported Outcomes Measures developed by ISPOR Translation and Cultural Adaptation group (TCA group) [18]. In details the translation process included following steps: preparation; forward translation; reconciliation; back translation; back translation review; harmonization; cognitive debriefing; review of cognitive debriefing results and finalization; proofreading; and final report. Permission to translate the DFS-SF into Polish was obtained in advance from the Mapi Research Trust (Lyon, France). Assessment of the reliability and validity of Polish DFS-SF was performed in native Polish patients with current DFU. Patients were recruited from a survey in the population of diabetic patients with active foot ulcers who were treated in ambulatory settings at the Department of Gastroenterology and Metabolic Diseases of the Medical University of Warsaw. As described in detail previously in our study on indirect costs associated with DFS in Poland (the participants overlap between the two studies) data on patients’ clinical condition, i.e., the duration of ulceration, diabetes type, the duration of diabetes and the duration of current treatment as well as basic demographic data, including age, gender, education, place of residence and employment sector were collected [19]. All questionnaires were self-administered and oral informed consent have been obtained from the participants (completed questionnaires documents participant consent). All data were collected and analyzed anonymously. Study was design as a non-interventional survey and Medical University of Warsaw ethics committee based on article 37al Pharmaceutical Law of 6 September (JL No, 126, item 1381) consolidated text of 27 February 2008 (JL No. 45, item 271) granted an exemption from requiring ethics approval [20, 21]. The severity of ulcers was evaluated using the PEDIS scale (Perfusion, Extent, Depth, Infection and Sensation classification system and score in patients with diabetic foot ulcer) designed by the International Working Group on the Diabetic Foot (IWGDF) [22]. The SF-36v2 scale was used to validate the DFS-SF measures, since SF-36 is considered a gold standard for measuring QoL including diabetes and its complication and has been previously used for DSF-SF validation [15, 17]. Permission to use Polish SF-36v2 and scoring software (QualityMetric Health Outcomes™ Scoring Software 4.5.1) was obtained from the QualityMetric Inc. (Lincoln, RI, USA).

The DFS-SF subscales scores were computed based on scoring conventions published elsewhere [15]. In details, the raw item scores were reverse coded so that the minimum possible score represented the worst quality of life, and the maximum possible score represented the best quality of life. Therefore, items were aggregated within each six subscales and then transformed to a score from 0 to 100, with higher scores indicating better quality of life for each subscale. Subscale Scores were calculated when less than 50% of the items for that subscale were missing. The missing responses were replaced by the mean of the item responses in the scale.

Acceptability (quality of data) of Polish DFS-SF were assessed by completeness of data and score distributions. We assumed that quality of data will be acceptable based on following criteria: i) missing data for summary scores <5%, ii) even distribution of endorsement frequencies across response categories and iii) floor/ceiling effects for summary scores <10% [23].

Item convergent validity was assessed by calculating the corrected item-scale correlations, i.e. the Spearman rank correlation between an item and the score of its hypothesized scale after removing the item. For each subscale, the item convergent validity was computed as the percentage of its items with corrected item-scale correlation of at least 0.6, consistent with a strong correlation in social science [24]. The item discriminant validity was computed as the percentage of items whose corrected item-scale correlation was greater than the correlation with other subscales of DFS-SF. Internal consistency of each subscale was examined using the Cronbach’s alpha coefficient. A Cronbach’s alpha coefficient value of greater than 0.70 was considered acceptable for the use of multi-item scales in conducting comparisons between groups [25].

Criterion validity was examined by Spearman’s rank correlation coefficient between DFS-SF and SF-36v2. Differential item functioning (DIF), i.e. excess correlation of a background characteristic with an item, beyond the association of the item with the score, was tested with ordinal logistic regression. We tested if background characteristics (sex, age, place of residence, education, type of diabetes, time from diagnosis of diabetes), when added to the baseline model explaining the item by the score, were significant as explanatory variables (calculations done in R, using chi-square statistic).

Correlations between severity of foot ulceration measured with the PEDIS scale or ulcer diameter and HRQoL were examined by Spearman’s rank correlation coefficient (rho). Hypothesis testing for differences between HRQoL in groups with different severity of foot ulceration was conducted using non-parametric tests, including the Mann Whitney-*U* test (to compare two groups) or the Kruskal-Wallis test (to compare more than two groups). The significance level in null hypothesis testing was set to 5% (α = 0.05). Statistical calculations were conducted using StatSoft, Inc. (2011) STATISTICA (data analysis software system), version 10. Tulsa, Oklahoma, USA, R version 3.3.2 Copyright (C) 2016 The R Foundation for Statistical Computing and Microsoft Office Excel 2010.

## Results

During the translation process, we did not modify any items but one major modification to DFS-SF questionnaire was made in order to improve the readability of the DFS-SF in Poland. Since some items in Polish have more elaborated descriptions, and because of the blurred vision in most DFU patients, we decided to use landscape (horizontal) instead of portrait (vertical) orientation of questionnaire. This allowed us to maintain enlarged fonts and made the questionnaire more readable (see Additional file 1: Appendix 1).

The DFS-SF validation study involved 212 patients diagnosed with DFU, with 4.4 years of DFU duration on average. Men (72%), residents of urban areas (79%) and type 2 diabetes patients (86%) prevailed, with 17.8 years representing the mean time since diagnosis. The mean population age was 62.5 years. More than 50% of patients had no perfusion abnormalities in the affected limb, and approximately 40% had a superficial full-thickness ulcer, generally without clinical symptoms of generalized infection. In the vast majority of patients (89%), loss of protective sensation was present. The average ulcer size was 5.5 sq. cm, and generally only one limb was affected. Detailed demographic and clinical characteristics of the patient population are presented in Table 1.

**Table 1:** Demographic and clinical characteristics of patients

Male, [n (%)] *N* = 212		153 (72.2%)
Age [mean (SD)] *N* = 210		62.5 (10.4)
Place of residence [n (%)] *N* = 211	Rural area	45 (21.3%)
	Urban area of <100 thousand.	54 (25.6%)
	Urban area of 100–500 thousand.	14 (6.6%)
	Urban area of more than 500 thousand.	98 (46.4%)
Education [n (%)] *N* = 212	Primary	36 (17.0%)
	Secondary	138 (65.1%)
	Higher	38 (17.9%)
Type of diabetes [n (%)] *N* = 211	Type 1	27 (12.8%)
	Type 2	181 (85.8%)
	Other	3 (1.4%)
Time (years) from diagnosis of diabetes [mean (SD)] *N* = 209		17.8 (11.6)
Time (years) from diagnosis of DFU [mean (SD)] *N* = 210		4.4 (4.7)
Time (weeks) of actual ulcer treatment [mean (SD)] *N* = 212		52.1 (99.0)
Size of ulcers in sq. cm [mean (SD)] *N* = 203		5.5 (10.3)
Perfusion [n (%)] *N* = 207	Grade 1	110 (53.1%)
	Grade 2	85 (41.1%)
	Grade 3	12 (5.8%)
Depth/tissue loss [n (%)] *N* = 210	Grade 1	84 (40.0%)
	Grade 2	81 (38.6%)
	Grade 3	45 (21.4%)
Infection [n (%)] *N* = 210	Grade 1	97 (46.2%)
	Grade 2	64 (30.5%)
	Grade 3	46 (21.9%)
	Grade 4	3 (1.4%)
Sensation [n (%)] *N* = 210	Grade 1	23 (11.0%)
	Grade 2	187 (89.0%)
Number of limbs affected [n (%)] *N* = 191	One	184 (96.3%)
	Both	7 (3.7%)

Quality of data were acceptable — missing data for summary scores were <5% for almost all items (see Additional file 2: Appendix 3). DFS-SF is a 5-point Likert-type scale with minimum possible score (1) represented the best quality of life and the maximum possible score (5) represented the worst quality of life. Given the nature of question items we can divide 5-point Likert-type scale to two positive, two negative and one neutral responses. The distribution of between positive (scores 1 and 2) and negative (scores 4 and 5) categories for all 29 items combined indicate no balance between positive (25.3%) and negative (52.8%) responses (see Additional file 2: Appendix 3). Uniform distribution would provide a mean percentage frequency of 20% for each of the 5 categories. As presented in figure in Additional file 2: Appendix 3, percentage frequency of positive responses (scores 1 and 2) were well below this value. In contrast, relatively high percentage frequency responses for score 4 was observed. The results of the Chi square tests indicate that the frequency distribution of responses amongst the 5 categories was not uniform. Indeed when comparing floor/ceiling effects for summary scores a relatively high floor percentage was found in the ‘leisure’ subscale (16.2%). None of the other subscales reached 10% of their floor percentage. However, the ceiling percentage was also low in all subscales of the Polish DFS-SF. It should be notated that in three subscales: ‘worried about ulcers/feet’, ‘negative emotions’ and ‘bothered by ulcer care’ none of the patients scored at the maximum level. A summary of result is provided in Table 2.

**Table 2:** Polish Diabetic Foot Ulcer Scale - Short Form results in patients with active diabetic foot ulcer

Subscale of DFS-SF	Mean	Median	SD	Floor %	Ceiling %	Cronbach’s alpha	
						Polish	English
Leisure	35.4	25.0	29.3	16.2%	2.5%	0.93	0.90
Physical health	43.5	45.0	22.6	5.4%	0.5%	0.87	0.86
Worried about ulcers/feet	41.7	41.7	23.8	3.4%	0.0%	0.92	0.84
Dependence/daily life	47.7	50.0	29.3	7.8%	2.9%	0.90	0.88
Negative emotions	34.8	30.0	23.3	9.8%	0.0%	0.89	0.93
Bothered by ulcer care	38.1	37.5	24.1	6.6%	0.0%	0.82	0.80

The internal consistency of all subscales of the Polish DFS-SF was high (Cronbach’s alpha ranged from 0.83 to 0.94) and comparable to those in English DFS-SF — see Table 2.

Item convergent validity was satisfactory — all but one (item 4I in the ‘negative emotions’ subscale) corrected item-scale correlations were >0.6 and median corrected item-scale correlation ranged from 0.61 to 0.81 — see Table 3 and Additional file 3: Appendix 2. Also item discriminant validity was satisfactory — the vast majority of items corrected correlations with the scale were greater than the correlations with other scales — see Table 3 and Additional file 3: Appendix 2. However there were some exception e.g. — item 4I in the ‘worried about ulcers/feet’ subscale had a corrected item-scale correlation of 0.63 but had a correlation of 0.73 with the ‘negative emotions’ subscale. This was expected since this item is shared in both those subscale and item 4I in the ‘negative emotions’ subscale had a corrected item-scale correlation of 0.54 but had a correlation of 0.75 with the ‘worried about ulcers/feet’ subscale. The ‘bothered by ulcer care’ subscale had the lowest item discriminant validity. Item - 5D in this subscale had a corrected item-scale correlation of 0.61 but had a correlation of 0.67 with the ‘dependence/daily life’ subscale.

**Table 3:** Item convergent and discriminant validity of the Polish Diabetic Foot Ulcer Scale - Short Form

Subscale of DFS-SF	Items (n)	Item convergent validity^a^ (%)	Item discriminant validity^b^ (%)	Median corrected item-scale correlation (range)
Leisure	5	100%	100%	0.81 (0.80 – 0.84)
Physical health	5	100%	100%	0.70 (0.64 – 0.74)
Worried about ulcers/feet	6	100%	83%	0.77 (0.63 – 0.81)
Dependence/daily life	5	100%	100%	0.81 (0.66 – 0.83)
Negative emotions	5	80%	80%	0.77 (0.54 – 0.79)
Bothered by ulcer care	4	100%	75%	0.61 (0.61 – 0.68)

^a^ Percentage of items in a scale whose corrected correlation with the scale was >0.6

^b^ Percentage of items in a scale whose corrected correlation with the scale was greater than the correlation with other scales

No DIF was found in most DFS-SF items. In only three cases did the demographic characteristics impact the item in a statistically significant way (see Additional file 4: Appendix 4): place of residence for item 2E (‘pain during night’), sex for item 3C (‘depend on others to get out of the house’), and both age and time from diagnosis of diabetes for item 5C (‘bothered by appearance of ulcer’). Because in total we had 29 items and 6 background characteristics tested (and so multiply hypotheses), we conclude there is no problem with DIF, i.e. with members of various subgroups interpreting items differently.

There were moderate associations among the Polish DFS-SF subscales, with a high positive correlation (0.82) between ‘worried about ulcers/feet’ and ‘negative emotions’ — see Table 4. This is not a surprise since those subscales share common item (4I) and both are related to patients’ emotions.

**Table 4:** Scale-scale correlations, according to the Spearman rank correlation coefficient — DFS-SF vs DFS-SF

	Leisure	Physical health	Worried about ulcers/feet	Dependence/daily life	Negative emotions	Bothered by ulcer care
Leisure	1.00	0.51	0.64	0.69	0.48	0.56
Physical health		1.00	0.64	0.66	0.54	0.56
Worried about ulcers/feet			1.00	0.64	0.82	0.72
Dependence/daily life				1.00	0.50	0.64
Negative emotions					1.00	0.63
Bothered by ulcer care						1.00

The Polish DFS-SF demonstrated good construct validity when correlated with the SF-36v2 — see Table 5. Although we did not identify DFS-SF subscales related to physical components (‘leisure’, ‘physical health’, ‘dependence/daily life’) to have significantly better correlation with physical component subscales of the SF-36v2 (i.e. ‘physical functioning’, ‘role physical’, and ‘bodily pain’) rather than mental component subscales of the SF-36v2 (i.e. ‘mental health’, ‘role emotional’, and ‘social functioning’), but similar regularities as for original (English) DFS-SF were observed i.e. strong correlation of DFS-SF ‘physical health’ and SF-36v2 ‘vitality’ (rho = 0.56) and ‘Bodily pain’ (rho = 0.63) [15]. Also, similar to original DFS-SF, Polish DFS-SF ‘dependence/daily’ was most highly related to the SF-36v2 ‘physical functioning’ (rho = 0.60) and ‘social functioning’ (rho = 0.58) subscales. Overall, Polish DFS-SF subscales showed stronger correlation with SF-36v2 subscales compared to the original DFS-SF, especially in ‘worried about ulcers/feet’ and ‘bothered by ulcer care’ subscales [15].

**Table 5:** Scale-scale correlations, according to the Spearman rank correlation coefficient — DFS-SF vs SF-36v2

	Leisure	Physical health	Worried about ulcers/feet	Dependence/daily life	Negative emotions	Bothered by ulcer care
Physical functioning	0.57	0.47	0.43	0.60	0.27	0.46
Role physical	0.59	0.38	0.46	0.54	0.34	0.46
Bodily pain	0.52	0.63	0.52	0.46	0.43	0.52
General health	0.41	0.46	0.52	0.39	0.50	0.43
Vitality	0.45	0.56	0.50	0.42	0.37	0.45
Social functioning	0.56	0.53	0.57	0.58	0.41	0.54
Role emotional	0.55	0.41	0.54	0.49	0.41	0.42
Mental health	0.46	0.55	0.60	0.41	0.48	0.48

Weak but significant negative correlations were found between ulcer size and’bothered by ulcer care’ subscale of DFS-SF. Surprisingly significant correlations were not found between ulcer size and other DFS-SF subscales — see Table 6. Similarly none of correlations were significant for the comparison of ulcer size and SF-36v2 subscales — see Table 6. Weak or moderately significant (except for ‘bothered by ulcer care’) negative correlations were found between loss of perfusion loss and DFS-SF subscales. No correlations were found for comparing both ‘depth/tissue loss’ and ‘infection’ and DFS-SF subscales. Similar regularities but less pronounced were observed for correlations of severity of foot ulceration (PEDIS scale) and HRQoL measured with SF-36v2, however surprisingly weak but significant positive correlation was observed between ‘depth/tissue loss’ and ‘general health’ subscale of SF-36v2 — see Table 6. Patents with loss of sensation scored significantly higher in all DFS-SF and SF-36v2 subscales — see Table 6. However, this is not a surprise, given that the loss of sensation usually results with pain reduction.

**Table 6:** Spearman rank correlation coefficient (except for ‘sensation’) between severity of foot ulceration (PEDIS scale) and HRQoL measured with DFS-SF and SF-36v2

	Ulcer size	Perfusion	Depth/tissue loss	Infection	Sensation^a^
	DFS-SF				
Leisure	−0,066	−0.253*	−0.079	−0.011	0.001
Physical health	0,016	−0.287*	0.019	−0.005	<0.001
Worried about ulcers/feet	−0,003	−0.184*	−0.031	−0.019	0.001
Dependence/daily life	−0,094	−0.312*	−0.009	−0.009	<0.001
Negative emotions	−0,081	−0.225*	−0.055	−0.063	<0.001
Bothered by ulcer care	−0,154*	−0.126	−0.057	−0.109	0.002
	SF-36v2				
Physical functioning	−0,045	−0.259*	−0.153*	−0.089	0.000
Role physical	−0,132	−0.131	−0.097	−0.103	0.044
Bodily pain	−0,041	−0.193*	−0.069	−0.082	0.000
General health	0,138	−0.112	0.189*	0.135	0.003
Vitality	0,038	−0.232*	0.029	0.036	0.003
Social functioning	−0,052	−0.104	−0.008	−0.039	0.002
Role emotional	0,000	−0.189*	−0.005	0.013	0.007
Mental health	0,053	−0.143*	−0.002	−0.024	0.001

^*^ *p* < 0.05

^a^ Mann Whitney-*U* test

## Discussion

To our knowledge translated condition— and region-specific PROMs that assess HRQoL in patients with diabetic foot ulcer have not been available up to now in Poland and no comprehensive analysis of HRQoL in the population of patients with DFU in Poland has been previously analyzed. The Polish translation of DFS-SF is the second after the Chinese translation that has undergone a full linguistic validation process. The present study is the first to assess of HRQoL in Polish patients with DFU using condition— and region-specific PROMs - DFS-SF.

The Polish DFS-SF demonstrated good scaling properties and good validity. The median corrected item-scale correlations and the internal consistency was excellent and similar to that of the original English version [15]. Correlations with the SF-36 scales also supported the construct validity of the Polish DFS-SF but also showed that Polish patients worries about ulcer care and bothered by treatment are more pronounced than in the English or Chinese population [15, 17]. This comes well with Polish experts opinions which concluded that out-patient care of patients with DFU is underfunded, difficult to access and the condition of treatment is unsatisfactory (source: experts’ on DFU survey). It is worth noting that our survey on direct cost of treatment among patients with DFU showed that more than 2/3 of out-patients specialist consultations are conducted in private care causing significant financial burden for patients.

The Polish DFS-SF also demonstrated good psychometric performance. Study on the influence of severity of foot ulceration on HRQoL showed DFS-SF is a more sensitive instrument than SF-36v2 when correlated with severity of ulceration measured with the PEDIS scale. Although it is worth noticing very modest correlation of ulcers’ severity and HRQoL was identified. Better sensitivity of DFS-SF is expected since SF-36v2 is a generic questionnaire and it was previously suggested that SF-36 measures of HRQoL may be confounded by non-foot complications of diabetes [9, 12]. However it is worth mentioning that SF-36v2 not DFS-SF can be easily converted to utility score for the purpose of economic evaluation.

We also verified DIF presence, in a simple logistic regression approach. Reassuringly, only 3 out of 29 items (and out of 6 background variables tested) have significant DIF. However, due to small sample sizes and the fact that our study was not originally planned to test DIF, these findings require further development. To the best of our knowledge, DIF have not been previously analyzed in DFS-SF.

At last, it should be noted that we’ve observed significant unbalance between positive and negative responses that might suggest trend for scoring lower in Polish DFS-SF. Indeed, when compare to Chinese DFS-SF, Polish patients scored significantly lower in all six subscales [17]. These differences may be due to differences in patient characteristics (i.e. some Chinese patients had healed foot ulcer), but it also may arise from trends shown in Polish population to score QoL lower compare to other developed countries. These was observed in QoL measures with either generic, e.g. EuroQol 5-Dimensions (EQ-5D) or condition-specific PROMs, e.g. Multiple Sclerosis Impact Scale (MSIS-29) [26, 27]. Small, but still visible differences in scoring against Polish population compare to other developed countries (Spain, Finland), has been observed in QoL measures in the aging population performed with the World Health Organization Quality of Life Assessment instrument (WHOQOL-AGE) [28].

In conclusion, the newly translated Polish DFS-SF may be used to assess the impact of diabetic foot ulceration on HRQoL in Polish patients, however data from different countries should be compared with caution.

## Additional files

Additional file 1: Appendix 1. Diabetic Foot Ulcer Scale-Short Form. (PDF 351 kb)
Additional file 2: Appendix 3. Frequency of responses. (DOCX 28 kb)
Additional file 3: Appendix 2. Item correlation with other subscales and corrected item-scale correlation. (DOCX 17 kb)
Additional file 4: Appendix 4. Differential item functioning (DIF) detection. (DOCX 14 kb)

## Abbreviations

DFS: Diabetic Foot Ulcer Scale
DFS-SF: The short form of Diabetic Foot Ulcer Scale
DFU: Diabetic foot ulcer
DIF: Differential item functioning
EQ-5D: EuroQol 5-Dimensions
HRQoL: Health related quality of life
ISPOR TCA: International Society For Pharmacoeconomics and Outcomes Research Translation and Cultural Adaptation group
IWGDF: International Working Group on the Diabetic Foot
JL: Journal of Laws
MSIS-29: Multiple Sclerosis Impact Scale
PEDIS: Perfusion, Extent, Depth, Infection and Sensation classification system and score in patients with diabetic foot ulcer
PROMs: Patient reported outcome measures
QoL: Quality of life
SF-36: The 36-Item Short Form Health Survey
SF-36v2: The 36-Item Short Form Health Survey version 2
WHOQOL-AGE: The World Health Organization Quality of Life Assessment
