# Supplementary material for: Health related quality of life in patients with diabetic foot ulceration — translation and Polish adaptation of Diabetic Foot Ulcer Scale short form
Source: Health Qual Life Outcomes. 2017 Jan 21;15:15. doi: 10.1186/s12955-017-0587-y (PMC5251239; doi:10.1186/s12955-017-0587-y)
Supplement: Additional file 1: Appendix 1. — Diabetic Foot Ulcer Scale-Short Form. (PDF 351 kb) [file 12955_2017_587_MOESM1_ESM.pdf]

Diabetic Foot Ulcer Scale-Short Form

INSTRUKCJA:

Pytania poniżej dotyczą wpływu problemów wynikających z obecności owrzodzenia stopy na Pana/Pani codzienne życie oraz samopoczucie.

Proszę przeczytać uważnie każde z pytań i zastanowić się, w jakim stopniu dotyczą one Pana/Pani problemów związanych z owrzodzeniem stopy.

Proszę odpowiedzieć na każde z pytań otaczając kółkiem jedną z cyfr w każdej linii. Jeśli nie jest Pan/Pani pewna jednoznacznej odpowiedzi, proszę o podanie jednej, najlepszej odpowiedzi.

1. **Jak bardzo Pani/Pana problemy związane z owrzodzeniem stopy wpłynęły na:**					
	Wcale	Nieznacznie	Umiarko-wanie	Dość znacznie	Bardzo
a) zaprzestanie uprawiania hobby i ulubionej aktywności rekreacyjnej	1	2	3	4	5
b) zmianę rodzaju uprawianego hobby i ulubionej aktywności rekreacyjnej	1	2	3	4	5
c) zaprzestanie wyjazdów wakacyjnych lub weekendowych	1	2	3	4	5
d) konieczność wybrania innego sposobu spędzania wakacji lub wolnego czasu niż dotychczas preferowany	1	2	3	4	5
e) konieczność poświęcenia dodatkowego czasu na zaplanowanie i organizację wolnego czasu	1	2	3	4	5

**2. W związku z problemami wynikającymi z owrzodzenia stopy, jak często Pani/Pan odczuwał/-a:**					
	Nigdy	Rzadko	Czasami	Często	Cały czas
a) osłabienie lub zmęczenie	1	2	3	4	5
b) wyczerpanie	1	2	3	4	5
c) trudności z zasypianiem	1	2	3	4	5
d) ból podczas chodzenia lub stania	1	2	3	4	5
e) ból w nocy	1	2	3	4	5

3. **Jak często z powodu owrzodzenia stopy Pani/Pan:**					
	Nigdy	Rzadko	Czasami	Często	Cały czas
a) wymaga pomocy innych osób w zakresie samoobsługi (np. mycia i ubierania się)	1	2	3	4	5
b) wymaga pomocy innych osób przy wykonywaniu prac domowych, takich jak: gotowanie, sprzątanie lub pranie	1	2	3	4	5
c) wymaga pomocy innych osób aby wyjść z domu	1	2	3	4	5
d) musi poświęcić więcej czasu na zaplanowanie i organizację codziennego życia	1	2	3	4	5
e) czuje, że wykonywanie jakichkolwiek czynności zajmuje więcej czasu niż chciałaby/chciałby Pani/Pan na to przeznaczyć	1	2	3	4	5

4. **Z powodu problemów związanych z owrzodzeniem stopy czuje Pani/Pan:**					
	Wcale	Niewielką	Umiarko-waną	Dość znaczną	Skrajną
a) złość, ponieważ nie może robić tego, co chce	1	2	3	4	5
b) frustrację z powodu tego, że inne osoby wykonują czynności, które Pani/Pan wolałaby/wolałby wykonać samodzielnie	1	2	3	4	5
c) frustrację z powodu tego, że nie jest w stanie zrobić tego co chce	1	2	3	4	5
d) obawę, że owrzodzenie nigdy się nie zagoi	1	2	3	4	5
e) obawę, że może być wykonana amputacja	1	2	3	4	5
f) obawę, że dojdzie do urazu stopy	1	2	3	4	5
g) depresję, ponieważ nie może robić tego, na co ma ochotę	1	2	3	4	5
h) obawę o wystąpienie owrzodzenia w przyszłości	1	2	3	4	5
i) złość, że to się Panu/Pani przytrafiło	1	2	3	4	5
j) frustrację z powodu problemów z poruszaniem się	1	2	3	4	5

5. Z powodu problemów wynikających z owrzodzenia stopy, jak często przeszkadza Pani/Panu:					
	Nigdy	Rzadko	Czasami	Często	Cały czas
a) odciążenie stopy z owrzodzeniem	1	2	3	4	5
b) ilość czasu poświęcanego na pielęgnację owrzodzenia stopy (w tym np.: zmianę opatrunków, oczekiwanie na wizytę u pielęgniarki/lekarza, oczyszczanie owrzodzenia)	1	2	3	4	5
c) wygląd, nieprzyjemny zapach oraz wyciek z owrzodzenia	1	2	3	4	5
d) zależność od innych osób w zakresie opieki nad owrzodzeniem stopy	1	2	3	4	5

Dziękujemy bardzo za wypełnienie ankiety!
