# Supplementary material for: Health related quality of life in patients with diabetic foot ulceration — translation and Polish adaptation of Diabetic Foot Ulcer Scale short form
Source: Health Qual Life Outcomes. 2017 Jan 21;15:15. doi: 10.1186/s12955-017-0587-y (PMC5251239; doi:10.1186/s12955-017-0587-y)

Appendix 3. Frequency of responses

| DFS-SF item no. | Description | The percentage of missing values for the items | The percentage frequency of responses | | | | |
| --- | --- | --- | --- | --- | --- | --- | --- |
|  |  |  | 5 | 4 | 3 | 2 | 1 |
| Q1A | Stopped from doing recreational activities | 2,9% | 34,2% | 30,7% | 16,6% | 8,5% | 10,1% |
| Q1B | Changed kinds of recreational activities | 4,9% | 31,8% | 30,8% | 15,4% | 10,3% | 11,8% |
| Q1C | Stopped from getting away for a holiday | 3,9% | 33,0% | 27,9% | 17,3% | 8,6% | 13,2% |
| Q1D | Made you choose different kind of holiday | 4,9% | 28,7% | 29,7% | 21,5% | 9,2% | 10,8% |
| Q1E | Had to spend more time planning leisure activities | 4,4% | 25,5% | 27,0% | 25,5% | 11,7% | 10,2% |
| Q2A | Felt fatigued | 1,0% | 12,8% | 42,9% | 30,5% | 12,3% | 1,5% |
| Q2B | Felt drained | 3,9% | 8,6% | 34,0% | 31,5% | 18,3% | 7,6% |
| Q2C | Had difficulty sleeping | 1,5% | 13,9% | 22,3% | 24,8% | 26,7% | 12,4% |
| Q2D | Pain while walking or standing | 1,0% | 27,6% | 34,0% | 21,2% | 11,8% | 5,4% |
| Q2E | Pain during night | 2,0% | 10,4% | 19,4% | 35,8% | 19,4% | 14,9% |
| Q3A | Depend on others to look after you | 0,5% | 14,7% | 18,6% | 19,6% | 22,5% | 24,5% |
| Q3B | Depend on others to do household chores | 0,5% | 24,0% | 21,6% | 18,1% | 15,2% | 21,1% |
| Q3C | Depend on others to get out of the house | 0,0% | 22,9% | 8,8% | 12,7% | 21,0% | 34,6% |
| Q3D | Spend more time planning daily life | 0,5% | 20,1% | 25,0% | 25,0% | 19,6% | 10,3% |
| Q3E | Felt doing anything took longer than would have liked | 1,5% | 26,2% | 34,2% | 23,3% | 12,4% | 4,0% |
| Q4A | Angry because not able to do what wanted | 1,0% | 6,9% | 45,8% | 25,1% | 17,7% | 4,4% |
| Q4B | Frustrated by others doing things for you | 1,5% | 10,4% | 37,6% | 25,2% | 18,3% | 8,4% |
| Q4C | Frustrated because not able to do what wanted | 1,5% | 12,4% | 42,6% | 23,3% | 15,8% | 5,9% |
| Q4D | Worried that ulcer will never heal | 1,5% | 21,3% | 41,6% | 18,3% | 12,9% | 5,9% |
| Q4E | Worried that you may have to have an amputation | 2,9% | 26,1% | 37,2% | 18,1% | 9,5% | 9,0% |
| Q4F | Worried about injury to feet | 2,4% | 20,5% | 45,5% | 17,0% | 13,0% | 4,0% |
| Q4G | Depressed because not able to do what wanted | 1,5% | 10,4% | 27,2% | 25,2% | 22,8% | 14,4% |
| Q4H | Worried about getting ulcers in future | 2,0% | 16,4% | 51,2% | 19,4% | 10,9% | 2,0% |
| Q4I | Angry that this has happened to you | 1,0% | 21,2% | 35,0% | 20,2% | 17,7% | 5,9% |
| Q4J | Frustrated because have difficulty getting about | 0,5% | 22,1% | 41,7% | 17,6% | 12,3% | 6,4% |
| Q5A | Bothered by having to keep weight off foot ulcer | 6,8% | 29,3% | 33,5% | 18,8% | 15,2% | 3,1% |
| Q5B | Bothered by amount of time involved in caring for ulcer | 4,9% | 21,0% | 39,0% | 22,6% | 12,8% | 4,6% |
| Q5C | Bothered by appearance of ulcer | 5,4% | 16,0% | 24,7% | 24,7% | 23,2% | 11,3% |
| Q5D | Bothered by having to depend on others for care of ulcer | 5,4% | 30,9% | 22,7% | 22,7% | 16,5% | 7,2% |


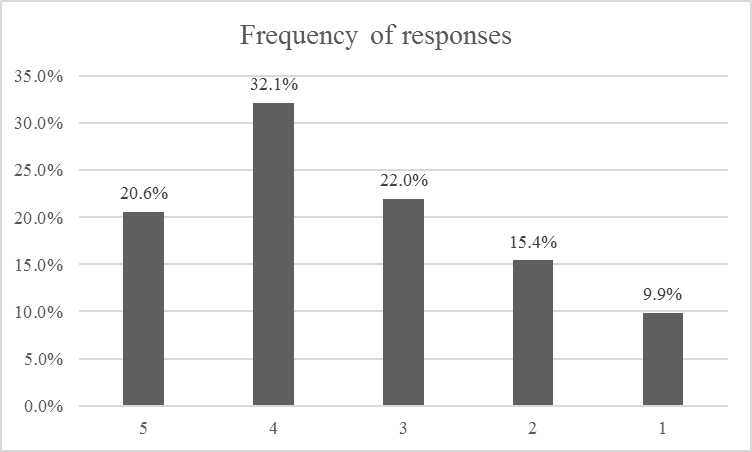

Supplement: Additional file 2: Appendix 3. — Frequency of responses. (DOCX 28 kb) [file 12955_2017_587_MOESM2_ESM.docx]
