# Supplementary material for: Health related quality of life in patients with diabetic foot ulceration — translation and Polish adaptation of Diabetic Foot Ulcer Scale short form
Source: Health Qual Life Outcomes. 2017 Jan 21;15:15. doi: 10.1186/s12955-017-0587-y (PMC5251239; doi:10.1186/s12955-017-0587-y)
Supplement: Additional file 3: Appendix 2. — Item correlation with other subscales and corrected item-scale correlation. (DOCX 17 kb) [file 12955_2017_587_MOESM3_ESM.docx]

Appendix 2. Item correlation with other subscales and corrected item-scale correlation.

| Subscale | DFS-SF item no. | Description | Correlation with other subscales | | | | | | Corrected item-scale correlation | | | | | |
| --- | --- | --- | --- | --- | --- | --- | --- | --- | --- | --- | --- | --- | --- | --- |
|  |  |  | Leisure | Physical health | Dependence/  daily life | Worried about ulcers/feet | Negative emotions | Bothered by ulcer care |  |  |  |  |  |  |
| Leisure | Q1A | Stopped from doing recreational activities |  | 0.508 | 0.543 | 0.549 | 0.374 | 0.492 | 0.811 | 0.854 | 0.905 | 0.915 | 0.906 |  |
|  | Q1B | Changed kinds of recreational activities |  | 0.403 | 0.532 | 0.560 | 0.410 | 0.511 | 0.857 | 0.809 | 0.901 | 0.905 | 0.907 |  |
|  | Q1C | Stopped from getting away for a holiday |  | 0.462 | 0.531 | 0.614 | 0.421 | 0.462 | 0.893 | 0.889 | 0.799 | 0.855 | 0.865 |  |
|  | Q1D | Made you choose different kind of holiday |  | 0.452 | 0.602 | 0.663 | 0.467 | 0.510 | 0.924 | 0.921 | 0.892 | 0.843 | 0.881 |  |
|  | Q1E | Had to spend more time planning leisure activities |  | 0.489 | 0.634 | 0.667 | 0.490 | 0.517 | 0.903 | 0.907 | 0.883 | 0.864 | 0.812 |  |
| Physical health | Q2A | Felt fatigued | 0.560 |  | 0.634 | 0.615 | 0.561 | 0.514 | 0.722 | 0.767 | 0.845 | 0.813 | 0.841 |  |
|  | Q2B | Felt drained | 0.572 |  | 0.698 | 0.606 | 0.600 | 0.554 | 0.797 | 0.735 | 0.841 | 0.854 | 0.867 |  |
|  | Q2C | Had difficulty sleeping | 0.323 |  | 0.423 | 0.482 | 0.334 | 0.370 | 0.813 | 0.790 | 0.640 | 0.830 | 0.783 |  |
|  | Q2D | Pain while walking or standing | 0.418 |  | 0.519 | 0.543 | 0.426 | 0.491 | 0.787 | 0.809 | 0.827 | 0.649 | 0.778 |  |
|  | Q2E | Pain during night | 0.292 |  | 0.423 | 0.480 | 0.382 | 0.399 | 0.847 | 0.838 | 0.813 | 0.820 | 0.700 |  |
| Dependence/  daily life | Q3A | Depend on others to look after you | 0.460 | 0.544 |  | 0.510 | 0.454 | 0.595 | 0.656 | 0.790 | 0.799 | 0.809 | 0.798 |  |
|  | Q3B | Depend on others to do household chores | 0.640 | 0.518 |  | 0.532 | 0.353 | 0.543 | 0.895 | 0.813 | 0.902 | 0.903 | 0.901 |  |
|  | Q3C | Depend on others to get out of the house | 0.569 | 0.558 |  | 0.541 | 0.418 | 0.531 | 0.896 | 0.891 | 0.808 | 0.890 | 0.902 |  |
|  | Q3D | Spend more time planning daily life | 0.643 | 0.620 |  | 0.595 | 0.484 | 0.553 | 0.918 | 0.902 | 0.888 | 0.832 | 0.872 |  |
|  | Q3E | Felt doing anything took longer than would have liked | 0.627 | 0.606 |  | 0.607 | 0.484 | 0.544 | 0.850 | 0.840 | 0.854 | 0.812 | 0.762 |  |
| Worried about ulcers/feet | Q4A | Angry because not able to do what wanted | 0.538 | 0.603 | 0.547 |  | 0.629 | 0.602 | 0.761 | 0.825 | 0.824 | 0.840 | 0.834 | 0.840 |
|  | Q4B | Frustrated by others doing things for you | 0.634 | 0.565 | 0.596 |  | 0.664 | 0.569 | 0.867 | 0.810 | 0.847 | 0.896 | 0.895 | 0.886 |
|  | Q4C | Frustrated because not able to do what wanted | 0.578 | 0.550 | 0.572 |  | 0.601 | 0.514 | 0.840 | 0.816 | 0.770 | 0.858 | 0.874 | 0.850 |
|  | Q4G | Depressed because not able to do what wanted | 0.553 | 0.569 | 0.574 |  | 0.695 | 0.621 | 0.857 | 0.859 | 0.859 | 0.744 | 0.847 | 0.835 |
|  | Q4I | Angry that this has happened to you | 0.344 | 0.354 | 0.344 |  | 0.728 | 0.571 | 0.760 | 0.772 | 0.782 | 0.758 | 0.631 | 0.755 |
|  | Q4J | Frustrated because have difficulty getting about | 0.609 | 0.633 | 0.600 |  | 0.744 | 0.673 | 0.860 | 0.861 | 0.861 | 0.841 | 0.849 | 0.790 |
| Negative emotions | Q4D | Worried that ulcer will never heal | 0.412 | 0.467 | 0.668 | 0.428 |  | 0.513 | 0.768 | 0.856 | 0.869 | 0.868 | 0.870 |  |
|  | Q4E | Worried that you may have to have an amputation | 0.412 | 0.468 | 0.647 | 0.414 |  | 0.509 | 0.867 | 0.791 | 0.848 | 0.855 | 0.918 |  |
|  | Q4F | Worried about injury to feet | 0.386 | 0.496 | 0.631 | 0.384 |  | 0.512 | 0.870 | 0.836 | 0.778 | 0.855 | 0.893 |  |
|  | Q4H | Worried about getting ulcers in future | 0.474 | 0.524 | 0.678 | 0.436 |  | 0.520 | 0.848 | 0.825 | 0.826 | 0.770 | 0.840 |  |
|  | Q4I | Angry that this has happened to you | 0.344 | 0.354 | 0.751 | 0.344 |  | 0.571 | 0.741 | 0.763 | 0.748 | 0.732 | 0.542 |  |
| Bothered by ulcer care | Q5A | Bothered by having to keep weight off foot ulcer | 0.568 | 0.489 | 0.625 | 0.575 | 0.536 |  | 0.677 | 0.821 | 0.839 | 0.836 |  |  |
|  | Q5B | Bothered by amount of time involved in caring for ulcer | 0.371 | 0.456 | 0.526 | 0.464 | 0.491 |  | 0.745 | 0.614 | 0.808 | 0.793 |  |  |
|  | Q5C | Bothered by appearance of ulcer | 0.372 | 0.421 | 0.542 | 0.373 | 0.531 |  | 0.831 | 0.834 | 0.608 | 0.831 |  |  |
|  | Q5D | Bothered by having to depend on others for care of ulcer | 0.486 | 0.468 | 0.594 | 0.674 | 0.466 |  | 0.811 | 0.823 | 0.828 | 0.615 |  |  |
