# Supplementary material for: Health related quality of life in patients with diabetic foot ulceration — translation and Polish adaptation of Diabetic Foot Ulcer Scale short form
Source: Health Qual Life Outcomes. 2017 Jan 21;15:15. doi: 10.1186/s12955-017-0587-y (PMC5251239; doi:10.1186/s12955-017-0587-y)
Supplement: Additional file 4: Appendix 4. — Differential item functioning (DIF) detection. (DOCX 14 kb) [file 12955_2017_587_MOESM4_ESM.docx]

Appendix 4. Differential item functioning (DIF) detection.

| Subscale | DFS-SF item no. | Description | p-value (Chi^2^) |
| --- | --- | --- | --- |
|  |  |  |  |
| Leisure | Q1A | Stopped from doing recreational activities | 0,734 |
|  | Q1B | Changed kinds of recreational activities | 0,475 |
|  | Q1C | Stopped from getting away for a holiday | 0,282 |
|  | Q1D | Made you choose different kind of holiday | 0,632 |
|  | Q1E | Had to spend more time planning leisure activities | 0,281 |
| Physical health | Q2A | Felt fatigued | 0,086 |
|  | Q2B | Felt drained | 0,269 |
|  | Q2C | Had difficulty sleeping | 0,631 |
|  | Q2D | Pain while walking or standing | 0,492 |
|  | Q2E | Pain during night | **0,035** |
| Dependence/  daily life | Q3A | Depend on others to look after you | 0,085 |
|  | Q3B | Depend on others to do household chores | 0,199 |
|  | Q3C | Depend on others to get out of the house | **0,020** |
|  | Q3D | Spend more time planning daily life | 0,210 |
|  | Q3E | Felt doing anything took longer than would have liked | 0,145 |
| Worried about ulcers/feet | Q4A | Angry because not able to do what wanted | 0,719 |
|  | Q4B | Frustrated by others doing things for you | 0,944 |
|  | Q4C | Frustrated because not able to do what wanted | 0,824 |
|  | Q4G | Depressed because not able to do what wanted | 0,149 |
|  | Q4I | Angry that this has happened to you | 0,238 |
|  | Q4J | Frustrated because have difficulty getting about | 0,563 |
| Negative emotions | Q4D | Worried that ulcer will never heal | 0,802 |
|  | Q4E | Worried that you may have to have an amputation | 0,490 |
|  | Q4F | Worried about injury to feet | 0,218 |
|  | Q4H | Worried about getting ulcers in future | 0,283 |
|  | Q4I | Angry that this has happened to you | 0,267 |
| Bothered by ulcer care | Q5A | Bothered by having to keep weight off foot ulcer | 0,081 |
|  | Q5B | Bothered by amount of time involved in caring for ulcer | 0,922 |
|  | Q5C | Bothered by appearance of ulcer | **0,016** |
|  | Q5D | Bothered by having to depend on others for care of ulcer | 0,097 |
